# Neural responses to visually observed social interactions

**DOI:** 10.1016/j.neuropsychologia.2018.02.023

**Published:** 2018-04

**Authors:** Jon Walbrin, Paul Downing, Kami Koldewyn

**Affiliations:** School of Psychology, Bangor University, UK

**Keywords:** Social interaction, Person perception, fMRI, Vision, Shapes, pSTS

## Abstract

Success in the social world requires the ability to perceive not just individuals and their actions, but pairs of people and the interactions between them. Despite the complexity of social interactions, humans are adept at interpreting those interactions they observe. Although the brain basis of this remarkable ability has remained relatively unexplored, converging functional MRI evidence suggests the posterior superior temporal sulcus (pSTS) is centrally involved. Here, we sought to determine whether this region is sensitive to both the presence of interactive information, as well as to the content of qualitatively different interactions (i.e. competition vs. cooperation). Using point-light human figure stimuli, we demonstrate that the right pSTS is maximally activated when contrasting dyadic interactions vs. dyads performing independent, non-interactive actions. We then used this task to localize the same pSTS region in an independent participant group, and tested responses to non-human moving shape stimuli (i.e. two circles’ movements conveying either interactive or non-interactive behaviour). We observed significant support vector machine classification for both the presence and type of interaction (i.e. interaction vs. non-interaction, and competition vs. cooperation, respectively) in the pSTS, as well as neighbouring temporo-parietal junction (TPJ). These findings demonstrate the important role that these regions play in perceiving and understanding social interactions, and lay the foundations for further research to fully characterize interaction responses in these areas.

## Highlights

• The pSTS is sensitive to visual dynamic social interactions.
• We show that the pSTS is sensitive to both the presence & contents of interactions.
• This effect is independent of face & body information.

## 1 Introduction

Social interactions are complex and dynamic and yet can quickly convey rich information about the actions, intentions, personality and goals of the participants involved. From an early age, people use the social interactions they observe to decide who to trust, who to learn from and who is in charge. Despite the importance of our ability to parse social interactions in building our knowledge of others and the relationships between them, relatively few studies have investigated the brain's response to observed social interactions between multiple actors. Instead, the bulk of the current ‘social vision’ literature has focused on the perception and appraisal of individual agents. One aspect of such work has greatly increased our understanding of the social brain and has implicated a core set of regions in perceiving and evaluating individual social objects (e.g. Downing et al., 2001; Kanwisher et al., 1997). What is not yet known is whether the processing of observed social interactions is similarly supported by focal, selective regions in the human brain.

A likely candidate in which to find such a region is the posterior superior temporal sulcus (pSTS). The pSTS has been described in the literature as the ‘hub’ of the social brain (Lahnakoski et al., 2012) and is often included in not only the network of areas involved in ‘person perception’, but also in the ‘action observation network’ and the ‘mentalizing network’ (Deen et al., 2015, Yang et al., 2015). In humans, converging functional magnetic resonance imaging (fMRI) findings implicate the pSTS (as well as neighbouring posterior superior temporal gyrus), as a region that may be sensitive to visually observed social interactions (Quadflieg and Koldewyn, 2017). Across a number of studies, univariate pSTS responses are greater when viewing videos of interactive behaviour relative to less interactive behaviour, especially within the right hemisphere. For example, interacting dyads > individual actions (Dolcos et al., 2012, Iacoboni et al., 2004), interacting dyads > non-interacting dyads (Centelles et al., 2011), and contingent interactions > ‘mirrored’ interactions – that is, two agents' actions that are contingent upon each other > the same synchronised action reflected and performed by both agents (Georgescu et al., 2014). Similarly, a few recent multivariate fMRI studies have also demonstrated sensitivity to interactive behaviour within the pSTS (Baldassano et al., 2017, Hafri et al., 2017). What's more, recent evidence in macaques also implicates the STS as a central region in the visual analysis of conspecific social interactions (Sliwa and Freiwald, 2017).

Interestingly, the pSTS response to social interactions does not seem to be dependent on perceiving *human* actors in these scenarios. Indeed, similar responses have been demonstrated with simple moving shape stimuli (i.e. self-propelled geometric shapes that create robust impressions of intentional behaviour) when contrasting interactions with other forms of shape motion; for example, greater pSTS activation is observed when viewing two shapes engaged in a complex interaction relative to shapes moving in an aimless, non-intentional manner (Castelli et al., 2000, Gobbini et al., 2007, Osaka et al., 2012, Tavares et al., 2007), or similar interactions vs. non-intentional, mechanical shape movement (Martin and Weisberg, 2003).

Importantly, however, most such paradigms have not sought to directly investigate social interaction *perception –* that is, observing interactions without a task that requires explicit judgements or inferences about agents’ behaviour. Across most of these studies, participants were required to make *explicit* theory-of-mind (ToM) judgements such as rating how ‘intentional’ shapes’ movements appeared to be (Castelli et al., 2000). Whilst *implicit* intentional processes are likely evoked when simply viewing moving shape displays (e.g. understanding the immediate purpose of an action), pSTS activation is observed when individuals make explicit intentional inferences, that is, *deliberative thinking or reasoning* about the contents of an individual's mind. The following extreme case demonstrates the influence of explicit inferences in the absence of socially meaningful behaviour: Lee et al. (2012) observed increased right pSTS activity when participants made explicit intentional attributions (i.e. detecting which shape chased another) based on random shape movement compared to non-social motion judgements. At present, it is unclear whether pSTS responses to abstract depictions of social interactions are evoked by the *presence* and *contents* of the social interaction itself (i.e. whether an interaction is taking place, and what is happening in an interaction, respectively), or if previously reported responses in the pSTS are driven by differences in animate motion or the task of making explicit social judgements.

Whilst the preceding evidence demonstrates that the pSTS is sensitive to visual interactive behaviour, much remains to be learned about what this region computes about such behaviour, especially qualitatively different interactions; for example, how might interactions in which two agents compete with each other be differentiated from those in which they cooperate with each other? Previous studies have found pSTS modulation when comparing qualitatively different interactions: Canessa et al. (2012) used still photographic stimuli and reported only minimal right pSTS activation when contrasting cooperative > affective interactions. Sinke et al. (2010) observed increased activation in the right pSTS for threatening interactions relative to teasing interactions; however, this difference was only observed when performing an orthogonal task (i.e. attending to the color of dots superimposed on the interaction), but not when performing an explicit inference task (e.g. identifying the emotional tone of the interaction) even though mean activation was greater during the inference task. These findings provide very preliminary evidence that the pSTS is sensitive to qualitative differences between otherwise visually similar interactions. One limitation of these studies is that they relied on univariate methods. In order to fully answer the question of whether the pSTS is sensitive to the actual *content* of an interaction rather than simply sensitive to the *presence* of an interaction, a multivariate approach may be required. Exploring content sensitivity in this fashion will provide clues to the functional role(s) of the pSTS in perceiving and understanding other people in interaction.

In experiment 1, we used point-light human stimuli across a large group of participants to demonstrate that the right pSTS is the most strongly activated region when contrasting interactions > visually matched non-interactions. In a separate group of participants, we then used the same contrast to localize human interaction-sensitive cortex within the pSTS before using a multivariate approach – that would afford greater sensitivity – to test whether this area would also contain information about the presence and qualitative content of abstract moving shape interactions. The task and stimuli were designed to focus on the role of pSTS in interaction perception while tightly controlling many ‘low-level’ visual cues and also attempting to reduce the influence of other social features known to engage this broad region. Specifically, these dynamic displays depicted highly-controlled moving shape stimuli that did not contain face or body information. Further, we used an orthogonal response task that did not require reporting interaction content, to minimize, as much as possible, explicit ToM inferences.

This approach allowed us to test two main hypotheses within the pSTS: Firstly, that above-chance classification of interactions vs. non-interactions would be observed. Secondly, we predicted that above-chance classification of different kinds of interactions. Specifically, we contrasted competitive vs. cooperative scenarios, as these types of interactions do not convey strongly differential emotional valence, which could confound interaction classification. A further practical advantage of testing these scenarios is that they are easily represented with moving shape animations. In the present study, for example, we conveyed cooperation via two shapes pushing the same side of an object together, and competition via two shapes pushing opposite sides of an object.

We then tested how *anatomically specific* such a pattern of results might be by comparing pattern classification performance in the localized interaction region to a neighbouring ToM-localized region in the temporo-parietal junction (TPJ). In addition, we also included a control region in the right lateral occipito-temporal cortex (LOTC) – an area centrally involved in action perception (Lingnau and Downing, 2015) – and recently shown to robustly classify between observed actions in the context of interactions, for example, different interactive actions (Hafri et al., 2017) and interactive compared to non-interactive actions (Wurm et al., 2017). Finally, we included additional univariate analyses to compare contrasts in the pSTS and TPJ with those from a recently published study that used similar stimuli (Isik et al., 2017).

## 2 Experiment 1: methods

### 2.1 Participants

Fifty participants (48 right-handed, 28 females, aged 19–34, *M* = 23.6 years, *SD* = 3.62) participated in the study. One participant's data was omitted from further analyses due to excessive head motion. The MIT Committee on the Use of Humans as Experimental Subjects reviewed and approved the experimental protocol and participants completed informed consent forms before taking part.

### 2.2 Paradigm

Participants viewed point-light dyads who either faced each other and were clearly engaged in a social interaction (e.g. both gesturing towards each other) or engaged in two independent actions (e.g. one riding a bike while the other walked). The independence of the two actions was further underscored by having the two figures face away from each other, and a line was placed down the center to form a ‘wall’ between the characters. The source of the interacting dyads was from Manera et al. (2010) and the source of the independent actions was from Vanrie and Verfaillie (2004). Individual videos ranged between 3 and 8 seconds (s) in length, but were blocked together to form 16 s blocks. The number of videos and the length of these videos was matched between conditions. Over the course of the scan session, 40 of the participants viewed 16 blocks of each condition, while the other 9 viewed only 8 blocks of each condition. Participants were instructed to simply maintain attention on the presented videos. A variety of other data (i.e. different data across participants) was collected in the same scan session as the currently described experiment, but will not be discussed further here.

Imaging data was acquired on a Siemens 3 T MAGNETOM Tim Trio Scanner at the Athinoula A. Martinos Imaging Center at MIT using a 32-channel head coil. Functional data were collected using a T2 * -weighted echo planar imaging (EPI) pulse sequence (TR = 2000 ms, TE = 30 ms, flip angle = 90°, FOV = 192 × 192 mm, matrix = 64 × 64 mm, slice width 3 mm isotropic, gap = .3 mm, 32 near-axial slices). In addition, a high resolution T1-weighted anatomical image (multi-echo MPRAGE) was collected (TR = 2530 ms, TE = 1.64 ms, 3.44 ms, 5.24 ms, 7.014 ms (combined with a RMS combination), echo spacing = 9.3 ms. TI = 1400 ms, flip angle = 7°, FOV = 220 × 220 mm, matrix size = 220 × 220 mm, slice thickness = 1 mm, 176 near axial slices, acceleration factor = 3, 32 reference lines).

### 2.3 Data analysis

All preprocessing steps and general linear modeling (GLM) was performed using Freesurfer version 5.3 (freesurfer.net). Preprocessing consisted of standard motion correction and then the alignment of each functional run to that participant's anatomical volume. Functional data were then smoothed using a 5 mm full width at half maximum (FWHM) Gaussian kernel. Smoothed data were used when defining regions of interest (ROIs), but percent signal-change data was extracted from unsmoothed data. For group-level analyses, data were normalized to the Freesurfer FSAverage template and a surface-based random effects group analysis was run across all participants, weighted by the amount of data contributed by each participant (i.e. the nine participants whose contrast maps were calculated from only two runs were weighted less heavily). All ROI analyses were performed in each participant's native anatomical space.

### 2.4 ROI definition and analysis

Subject-specific pSTS ROIs were created using a leave-one-out method (e.g. the ROI was defined by 3 runs of data and percent signal change was calculated from the left out fourth run). This process was iterated until percent signal change had been calculated from all runs. The nine participants who had only two runs of data to work with were not included in the percent signal change analysis. ROIs were defined by intersecting an 8 mm-radius sphere with the cluster peak (i.e. highest voxel t-value) in the right pSTS/STG where the contrast maps were thresholded at *p* < .005 uncorrected. Of the 40 participants entered in the ROI analysis, we could not localize an ROI for five participants, leaving 35 participants in the final ROI analysis.

## 3 Experiment 1: results

**Fig. 1:**
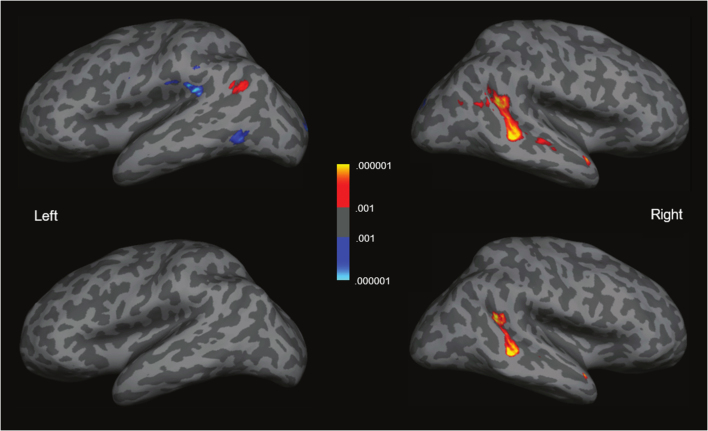
Group analysis for the social interaction > independent actions contrast (N = 49). The top panel is thresholded at *p* < .001 uncorrected while the bottom panel is FDR-corrected for multiple comparisons at 5%. The color-bar represents significance (*p*-value).

**Fig. 2:**
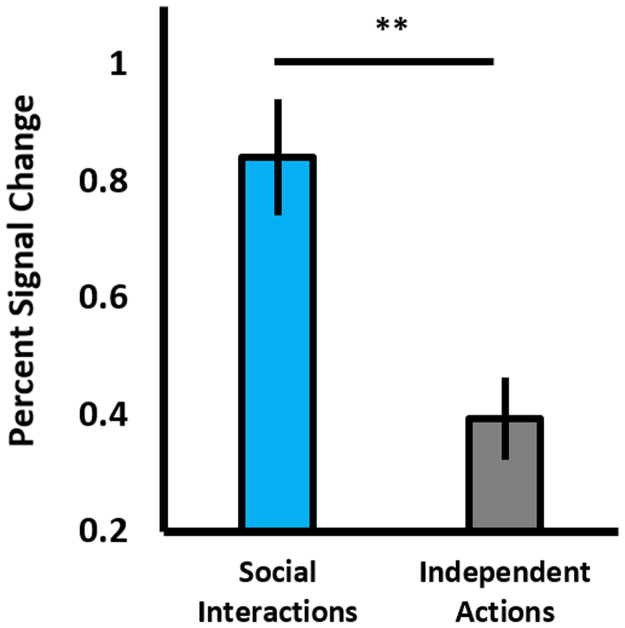
A bar chart showing mean percent signal change for the pSTS, for both social interactions and independent actions. ** = *p* ≤ .001. Error bars are SEM.

The group analysis showed a region within the right pSTS that responded much more strongly to social interaction than to independent actions. Indeed, when false discovery rate (FDR) corrected for multiple comparisons (at 5%), only two clusters remain – both in the STS (a large cluster in the pSTS and a small cluster in very anterior STS; see Fig. 1). Even when thresholded at a more liberal threshold of *p* < .001 *uncorrected*, this activation remains primarily in the STS, spreading more anteriorly on the right, and showing a small and much weaker response on the left (see Fig. 1). No other cortical region reached significance in this contrast. The ROI analysis revealed a significantly higher response to social interactions than independent actions (paired *t*-test, *t*(34) = 8.20, *p* < .001; see Fig. 2).

## 4 Experiment 2: methods

### 4.1 Participants

23 right-handed participants (12 females; aged 18 – 30, *M* = 22.43 years, *SD* = 3.07) were recruited from the Bangor University student population. Data from two participants were removed from all analyses due to consistently low behavioral response accuracy (i.e. < 50% accuracy across all runs). The study was authorized by the School of Psychology ethics committee, and participants gave informed consent and received monetary compensation for the session.

### 4.2 Stimuli, design, and task

**Fig. 3:**
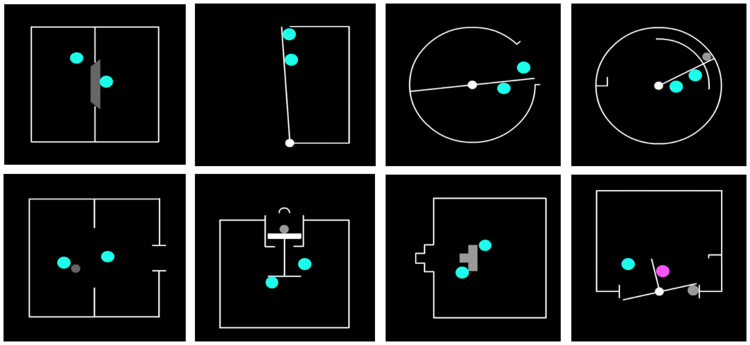
Each panel depicts one of the 8 different scenarios in which the two agents interacted via a ‘push-able’ object, as in the competition and cooperation conditions; the non-interaction conditions, by contrast, featured the same scenarios but the agents moved randomly within the scene without interacting with each other or moving the push-able object(s). The bottom right panel shows an example ‘color-change’ frame in which one of the agents momentarily changed color. (For interpretation of the references to color in this figure legend, the reader is referred to the web version of this article.)

The stimuli depicted eight different scenes from an aerial perspective (see Fig. 3; example videos can be viewed via supplementary materials, section F), each lasting 6 s. In each scene, two animate agents – blue circles – moved around a walled region in a self-propelled manner (visual angles: Agents = .80°; average walled space width = 7.22°). Each scenario contained a ‘push-able’ interaction object (e.g. a door) that served as the focus of the interaction. Eight experimental conditions were created from 2 × 2 factor levels: Interactive state (*interaction* and *non-interaction*) and interaction type (*competition* and *cooperation*). Interaction variants of competition and cooperation, respectively, depicted the shapes either working together, or against each other to achieve their respective or shared goals. To ensure that the outcome of each video was not confounded with the type of interaction, half of both interaction types resulted in successful object action (e.g. a door was successfully opened), and half resulted in unsuccessful action. Because successful and unsuccessful object actions had qualitatively different meanings for competition and cooperation (i.e. cooperative success results in fulfillment of both agents’ action goal, whereas competitive ‘success’ only applies to one of the agents), we did not analyze successful vs. unsuccessful object actions. To minimize the visual familiarity of individual videos, each video was presented at four 90° rotations. In total, there were 256 novel stimuli (i.e. 4 conditions x 2 outcomes x 8 scenarios x 4 video rotations), of which 240 were randomly selected for the experiment.

As the non-interaction variants of competition and cooperation did not contain interactive behaviour, the agents’ movements in these conditions were generated by scrambling the agents’ motion trajectories from their respective interaction conditions. This was achieved by separately splitting each agent's motion trajectory into 1 s segments, and rotating the direction of each segment before joining them into continuous paths. This gave the impression of animate and self-propelled movement, but ensured that agents did not appear to interact with each other or the ‘push-able’ object. To reduce the appearance that agents were ‘magically’ causing object movement, the motion paths of the push-able interaction objects were reversed for each video to de-correlate agent and object movement. Additionally, to ensure that large differences in the proximity of agents would not drive classification differences between conditions, we ensured that agents were confined to the same region of the display as in the interaction stimuli from which they were generated (e.g. if both agents interacted within one half of the display in a given interaction stimulus – for example, behind a closed door – we ensured that both agents’ movements were restricted to the same area in the non-interaction variant of that stimulus).

To ensure that stimulus motion energy did not differ between conditions, we used the same approach adopted in previous studies by computing differences in pixel intensity between video frames (e.g. Grezes et al., 2007). Specifically, we computed the average difference in pixel luminance between contiguous pairs of frames for each video, and then entered these values into a 2 × 2 ANOVA (i.e. interaction and non-interaction as levels of the first factor, with competition and cooperation as levels of the second factor). No term was significant (all *p*s > .462; see supplementary materials, section D) indicating no difference in motion energy between conditions. Therefore, neural response differences between conditions could not be attributed to differences in global stimulus motion between conditions.

A blocked-design was used in which 10 runs were completed, each lasting 242 s and consisted of eight experimental blocks, two per condition, 20 s each (3 × 6 s videos + 2 s fixation epoch at the end of the block), along with three 20 s rest blocks at the beginning, middle, and end of each run. A total of 80 experimental blocks were completed (i.e. 240 experimental trials, 20 blocks per condition, 60 stimuli per condition). Block ordering was randomized across runs, and runs were randomized across participants.

Participants performed an orthogonal response task whilst viewing stimuli that was intended to minimize the tendency to make explicit ToM attributions; participants pressed a button whenever one of the agents momentarily changed color (i.e. from blue to pink). This change lasted for one animation frame (i.e. 41.67 ms) and always occurred 5.5 s after trial onset. To minimize the predictability of these trials, only one trial per block contained a color-change, with presentation order randomized across blocks. The brevity of the color-change also served to encourage active attending to both agents, as this change could easily be missed if a participant's attention momentarily drifted away from the agents. While not cognitively demanding, this was a relatively difficult task; it required consistent attention to both agents in order to catch the very brief change.

### 4.3 Localizer stimuli, design, and tasks

To localize interaction-sensitive regions of the pSTS, we used a shortened version of the task used in experiment 1 (i.e. contrasting two interacting point-light figures with two figures performing independent actions). Participants completed two blocked runs (i.e. 11 × 18 s blocks – four blocks per condition along with three fixation blocks, resulting in 198 s run length), with blocks presented in a randomized order across runs. In order to localize ToM-sensitive cortex in the TPJ, participants also completed two runs of a written story false-belief localizer task (Dodell-Feder et al., 2011). The stimuli and protocol for this task are described elsewhere in detail (saxelab.mit.edu/superloc.php).

### 4.4 ROI definition

Subject-specific pSTS ROIs were created based on activation from the independent interaction localizer task with an uncorrected height threshold of *p* < .05. ROIs were defined by intersecting a 6 mm-radius sphere with the cluster peak (i.e. highest voxel t-value) in the right pSTS. As before, we chose to localize pSTS only in the right hemisphere due to observed stronger right lateralization in the group data in experiment 1, as well as in prior interaction studies (e.g. Georgescu et al., 2014). TPJ ROIs were created in an identical way (i.e. intersecting a 6 mm-radius sphere with right TPJ peak activation from the false belief > physical change contrast, with the same height threshold). Both pSTS and TPJ ROIs were successfully localized in most participants (i.e. 19 and 16 participants, respectively; see supplementary materials, Table C, for further details of omissions). The resulting ROIs did not overlap and pSTS ROIs were observed to be significantly more anterior and ventral than TPJ ROIs across all participants in which both ROIs were localized (see supplementary materials, section C). We defined the LOTC ROI by centering a 6 mm-radius sphere at the peak coordinates (MNI x, y, z: 54 − 58 − 10) for the whole brain interaction > non-interaction contrast from independent data (i.e. a pilot study that used similar moving shape stimuli; see supplementary materials, section A).

### 4.5 MRI acquisition parameters and pre-processing

Scanning was performed with a Philips 3T scanner at Bangor University. Functional images were acquired with the following parameters: a T2*-weighted gradient-echo single-shot EPI pulse sequence; TR = 2000 ms, TE = 30 ms, flip angle = 90°, FOV = 230 × 230 × 132 mm, acquisition matrix = 76 × 74 (reconstruction matrix = 128 × 128); 35 ascending slices (width = 3 mm, gap = .8 mm), acquired voxel size (mm) = 3.03 × 3.11 × 3.0 (reconstructed voxel size (mm) = 1.8 × 1.8 × 3.0). Four dummy scans were discarded prior to image acquisition for each run. Structural images were obtained with the following parameters: T1-weighted image acquisition using a gradient echo, multi-shot turbo field echo pulse sequence, with a five echo average; TR = 12 ms, TE = 3.5 ms − 10.2 ms, in 1.6 ms steps, acquisition time = 329 s, FA = 8°, FOV = 250 × 250 × 170, acquisition matrix = 252 × 224 (reconstruction matrix = 256 × 256); 170 contiguous slices, acquired voxel size = .99 × 1.12 × 2.0 mm (reconstructed voxel size = 1 mm^3^).

Before realignment, functional image runs were inspected using ArtRepair version 5b (cibsr.stanford.edu/tools/human-brain-project/artrepair-software.html**)** to assess excessive scan-to-scan head-motion. As head motion can reduce classification accuracy (Wutte et al., 2011), we adopted a strict motion threshold: Any participant runs containing > .5 mm scan-to-scan movement were omitted. Single runs were omitted from six participants, and three runs from one further participant (see supplementary materials, Table B, for full details of omissions). All pre-processing steps (i.e. realignment, co-registration, segmentation, normalization, & smoothing) were performed in SPM12 (fil.ion.ucl.ac.uk/spm/software/spm12). All default parameters were used except for a 6 mm FWHM Gaussian smoothing kernel.

### 4.6 Data analysis

GLM analysis was implemented on participants’ normalized data in SPM12. Block duration and onsets for each experimental condition were modelled using a boxcar reference vector and convolved with a canonical hemodynamic response function. Smoothed beta maps, and subsequent t-maps, were generated for the two localizer task contrasts, whilst unsmoothed beta maps were created for classification and univariate percent signal change (PSC) analyses. Four separate regressors (i.e. competition and cooperation, along with their respective non-interaction variants) were generated for each run. For the interaction vs. non-interaction contrasts, the corresponding regressors were modelled together, that is, interaction = [competition + cooperation], non-interaction = [non-interaction competition + non-interaction cooperation].

The Decoding Toolbox (Hebart et al., 2015) was used to implement linear support vector machine (SVM) classification (hyperparameter C = 1) with a leave-one-run-out scheme; for each classification fold, voxel ‘patterns’ of beta-estimates for each condition, for all but one run were used as training data, and performance was tested on data from the ‘left-out’ run. This was performed iteratively until all runs had been tested (i.e. 10 iterations for 10 runs). For each classification contrast, a mean accuracy value was generated for each ROI, per participant. This value was based on the correct classification of each condition (e.g. competition or cooperation), averaged across classification folds. For each ROI and contrast, accuracy values were entered into one-sample *t*-tests against chance (i.e. 100% / 2 classes = 50% chance). An FDR-correction threshold of *p* ≤ .01, was determined based on 5 *t*-tests yielding one-sided p-values ≤ .05 (i.e. .05 / 5 = .01). FDR corrected p-values of ≤ .025 were determined for significant *t*-tests that followed PSC ANOVAs, (i.e. .05 / 2 = .025).

Response accuracy (%) was calculated for each participant (i.e. a button-press response occurring < 1.5 s after color-change onset). Runs containing > 2 inaccurate responses (i.e. < 75% accuracy) were omitted from further analysis (i.e. 1 run from 2 separate participants) to ensure that only actively attended runs were included.

## 5 Experiment 2: results

### 5.1 Behavioral data

A 2 × 2 ANOVA was performed on response accuracy scores with interaction and non-interaction as levels of the first factor and competition and cooperation as levels of the second factor. Unexpectedly, a main effect between interaction and non-interaction was observed (interaction: *M* = 95.02, *SD* = 2.87; non-interaction: *M* = 91.70, *SD* = 7.03; *F*(1,20) = 6.36, *p* = .020), demonstrating that participants were more accurate in the interaction than non-interaction conditions. Neither the main effect between competition and cooperation (competition: *M* = 95.41, SD = 4.63; cooperation: *M* = 94.62, SD = 4.09; *F*(1,20) = .38, *p* = .543), nor the interaction term was significant (*F*(1,20) = 1.40, *p* = .251).

Additionally, we obtained stimulus ratings from a separate group of participants (N = 20) outside of the scanner. This served to aid our interpretation of what might drive greater response accuracy for interactions than non-interactions. Participants viewed a randomly selected subset of the original stimuli (i.e. after collapsing across the 4 rotation variants of each stimulus, there were 64 ‘unique’ stimuli; 32 of these were presented – 8 per condition, balanced across successful and unsuccessful outcome variants). Participants gave Likert-scale ratings (i.e. 1 = strongly disagree, 7 = strongly agree) of the videos, for the following three statements: ‘The agents interacted with each other’ (interactivity); ‘The agents were goal-directed’ (goal-directedness); ‘The agents were alive/animate’ (animacy). Along with interactivity, we included ratings of goal-directedness and animacy as these constructs are shown to drive pSTS responses in moving shape displays (e.g. Gao et al., 2012). We then entered participant ratings into three 2 × 2 ANOVAs, one for each question (see supplementary materials, section E, for full statistics). A main effect between interaction and non-interaction was observed for all three statements (all *ps* < .001), showing that interactions were perceived as more interactive, goal-directed, and animate than non-interactions. No other term was significant across any of the three ANOVAs (all *ps* > .100), indicating no perceived differences in interactivity, goal-directedness, or animacy between competition and cooperation (or the respective non-interaction variants of these conditions).

### 5.2 SVM classification analyses

**Fig. 4:**
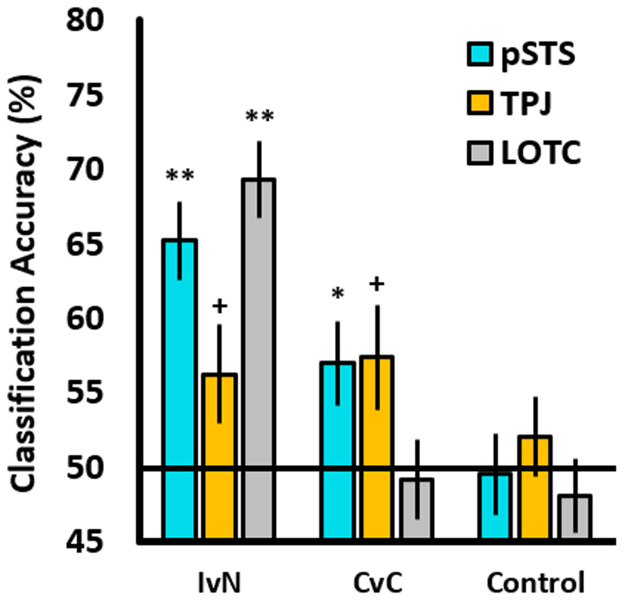
A bar chart showing mean SVM classification accuracy values (%) for each contrast. IvN = interaction vs. non-interaction; CvC = competition vs. cooperation; Control = control contrast. The horizontal line represents assumed chance-level classification (50%). ** = significant at *p* ≤ .001 level; * = significant at FDR-corrected level *p* ≤ .01; + = significant at uncorrected *p* ≤ .05 level. Error bars are SEM.

To test our main hypotheses – that the pSTS would significantly differentiate interactions from non-interactions, and competition from cooperation – we performed SVM classification within subjects’ pSTS ROIs (see Fig. 4). For both main contrasts, mean classification accuracy was significantly greater than chance (interaction vs. non-interaction: *M* = 65.20%, *SD* = 11.15, *t*(18) = 5.94, *p* < .001; competition vs. cooperation: *M* = 56.97%, *SD* = 11.15, *t*(18) = 2.53, *p* = .010). For the TPJ, we also observed above-chance classification for both contrasts, but at the uncorrected (*p* ≤ .05) level only (interaction vs. non-interaction: *M =* 56.24%, *SD* = 13.07, *t*(15) = 1.90, *p* = .038; competition vs. cooperation: *M =* 57.35%, *SD* = 13.80; *t*(15) = 2.13, *p* = .025). Therefore, above-chance classification for both contrasts was observed in the pSTS and also to a weaker extent in the TPJ.

We then compared these results to a control region in the LOTC; we observed above-chance classification in the interaction vs. non-interaction contrast (*M* = 69.29%, *SD* = 11.77, *t*(19) = 7.32, *p* < .001) but not for competition vs. cooperation (*M* = 49.16%, *SD* = 11.95, n.s). These results are consistent with recent studies that demonstrate robust classification between a variety of actions, such as pushing and pulling (Hafri et al., 2017), as well as between interactive and non-interactive actions (Wurm et al., 2017); strong classification in the interaction vs. non-interaction, but not competition vs. cooperation contrast likely reflects the differential amount of object-oriented action between conditions (i.e. the agents pushed objects in *both* interaction conditions, but did not in the non-interaction conditions).

Although we found no difference in motion energy between any contrasted pair of conditions (see section 4.2) we sought to confirm that no low-level stimulus confounds (e.g. differences in total velocity or motion energy) might account for competition vs. cooperation classification in the pSTS and TPJ. To this end, we trained a classifier on the corresponding non-interaction contrast that was matched for motion energy (i.e. non-interaction competition vs. non-interaction cooperation; see Fig. 4) and, as expected, found no significant classification in either region (both *ps* > .466).

#### 5.3 Univariate analyses

In addition to classification analyses, we also sought to determine whether univariate responses differed between pSTS and TPJ, for both main contrasts. Based on the univariate contrast from experiment 1, we expected greater activation for shape interactions than non-interactions. For the competition vs. cooperation contrast however, we had no clear expectation as to whether competition or cooperation should evoke greater activation, or whether such differentiation could be captured with univariate analysis. However, a similar contrast in a recent study (Isik et al., 2017) demonstrated a trend towards greater activation for ‘hindering’ compared to ‘helping’ moving shape interactions, as well as greater activation for interactions compared with ‘physical’ interactions (i.e. inanimate ‘billiard ball’ type movements), and so we sought to determine whether our data showed similar trends.

**Fig. 5:**
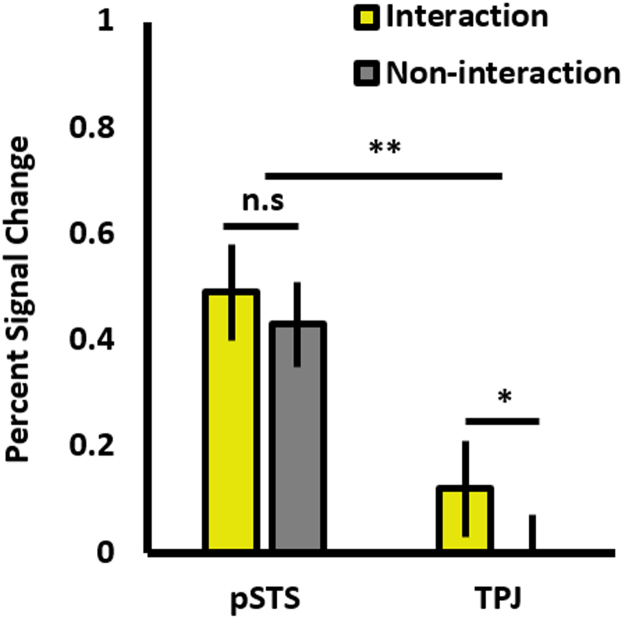
A bar chart showing mean percent signal change for the interaction and non-interaction conditions, for the pSTS and TPJ. ** = *p* = .003; * = significant at FDR-corrected level *p* ≤ .025. Error bars are SEM.

To this end, we extracted subjects’ PSC values for each condition and ROI, and ran two 2 × 2 ANOVAs – one for each of the contrasts (with ROI as the first factor, and the respective contrast conditions as levels of the second factor). For interaction vs. non-interaction (see Fig. 5), we observed a main effect of ROI (pSTS: *M* = .43, *SD* = .42; TPJ: *M* = .05, *SD* = .30; *F*(1,14) = 12.74, *p* = .003), a marginal main effect of contrast (interaction: *M* = .29, *SD* = .39; non-interaction: *M* = .20, *SD* = .34; *F*(1,14) = 4.26, *p* = .058), but no interaction between the two factors (*F*(1,14) = 1.88, *p* = .192). Paired *t*-tests (2-tailed) revealed a significant difference between interaction and non-interaction in the TPJ (*t*(15) = 2.53, *p* = .023), but unexpectedly, the same trend was not significant in the pSTS (*t*(18) = 1.59, *p* = .129).

**Fig. 6:**
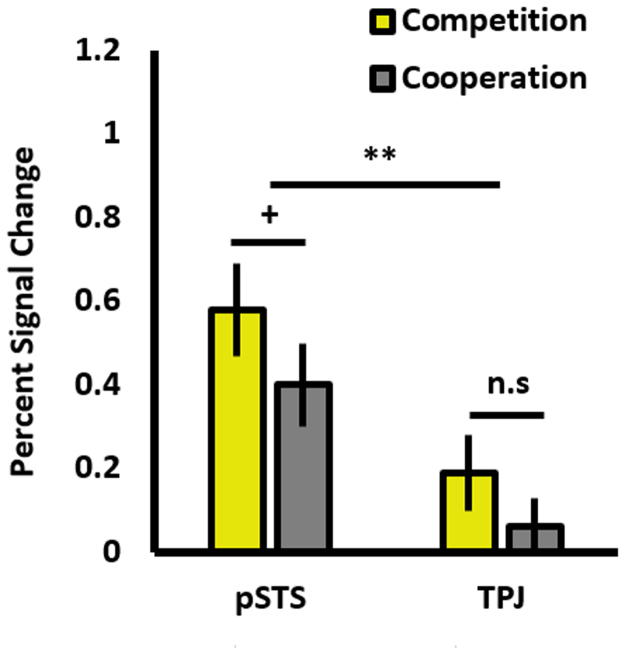
A bar chart showing mean percent signal change for the competition and cooperation conditions, for the pSTS and TPJ. ** = *p* = .008; + = significant at the uncorrected level *p* ≤ .05. Error bars are SEM.

For the competition vs. cooperation contrast (see Fig. 6), a main effect of ROI was observed (pSTS: *M* = .49, *SD* = .41; TPJ: *M* = .12, *SD* = .32; *F*(1,14) = 9.71, *p* = .008), along with a marginal main effect of contrast (competition: *M* = .37, *SD* = .48; cooperation: *M* = .21, *SD* = .35; *F*(1,14) = 3.61, *p* = .078), and marginal interaction (*F*(1,14) = 3.94, *p* = .067). Follow-up *t*-tests (2-tailed) revealed greater activation for competition than cooperation in the pSTS at the uncorrected level (*p* ≤ .05) only (*t*(18) = 2.22, *p* = .040), but not in the TPJ (*t*(15) = 1.68, *p* = .113).

## 6 Discussion

The results from experiment 1 reveal that a region in the right pSTS is strongly responsive to dyadic social interactions; it is one of only a few regions that shows such a response in a whole-brain group analysis in a relatively large sample, and it responds about twice as strongly to such interactions when compared to visually similar depictions of two individuals performing non-interactive actions. The identification of the pSTS as being uniquely responsive to human interactions is replicable in an independent set of data from our own lab (see supplementary materials, section A, and Supplementary fig. 1) as well as in data from a recent paper using similar stimuli (Isik et al., 2017). This result is not driven by aspects of human appearance known to drive pSTS responses (e.g. Deen et al., 2015): Our stimuli contained no face information, and the two conditions each contained two point-light figures moving biologically. Similarly, in experiment 2, where our stimuli contained interactions depicted by simple shapes, we observed significant results in the region of the pSTS functionally localized with human interactions: Above-chance SVM classification was observed when contrasting interaction and non-interaction stimuli, as well as when contrasting two qualitatively different types of interaction (competition and cooperation). These findings suggest the pSTS is involved not only in recognizing social interactions, but also in assessing the meaning and content of such social interactions (i.e. differentiating between different interactions). Whilst these results might suggest a unique selectivity for social interaction perception per se that cannot be attributed to simple differences in face or body information, there are several caveats that prevent such a clear and unequivocal interpretation.

Firstly, the univariate results in experiment 2 do not show the same trend as in experiment 1 – that is, the interaction > non-interaction contrast for our moving shape stimuli did not reach significance in the pSTS. Secondly, we observed greater response accuracy for interaction than non-interaction moving shape stimuli in the behavioral data suggesting that, against our expectations, attentional or explicit ToM-related differences between the interaction and non-interaction conditions are likely. Thirdly, independent ratings of the moving shape stimuli showed that interaction stimuli were perceived as significantly more goal-directed and animate than non-interactions, and therefore these sources of information likely contribute to pSTS responses to these conditions. It is worth noting, however, that these potentially confounding contributions did not drive univariate responses in the pSTS to be significantly greater for the interaction condition than the non-interactive condition in this dataset. They may be, however, a source for the difference we see in the TPJ between these two conditions. Nevertheless, given the complications in our data we cannot make any strong claims for interaction selectivity in the pSTS based on the interaction vs. non-interaction contrast in experiment 2 alone.

However, these limitations do not apply to the competition vs cooperation contrast in experiment 2. We observed significant classification and univariate results in the pSTS to this contrast that cannot be attributed to differences in response accuracy, independent stimulus ratings, or motion energy (i.e. these conditions were matched across these measures). Although there is mixed evidence as to whether the pSTS reliably differentiated the interaction > non-interaction contrast across our two stimulus sets, our univariate and classification results give direct evidence that the pSTS differentiates between qualitatively different interactions and suggest that the pSTS might play a central role in the perception and understanding of social interactions. Whilst further evidence is undoubtedly required to fully support this possibility (e.g. we cannot differentiate whether perceptual differences or potential qualitative differences in the interactors’ intentions might drive this effect), the present findings align closely with a very recent paper investigating a similar research question and using conceptually similar stimuli (Isik et al., 2017). Together, these two studies suggest a central role for a region in the pSTS during social interaction perception, however the precise role(s) will need to be further specified by future research.

We also found that the pSTS was not the only region to differentiate between our conditions; similar classification responses were observed in neighbouring ToM-task localized TPJ, although these responses were somewhat weaker. In addition, their relative responses to the univariate interaction > non-interaction contrast are similar, although the difference was only significant in the TPJ. It is, however, worth noting that in a similar contrast in Isik et al. (2017), as well as in pilot data we collected (see supplementary materials, section A), the interaction > non-interaction contrast *did* reach significance in the pSTS. Conversely, for the competition vs. cooperation contrast, similar differentiation of conditions was observed in both regions, but only reached (uncorrected) significance for the pSTS. Therefore, similarities in classification performance and mixed univariate results prevent a clear functional separation of the two regions.

In some ways, the similarity between the two regions is not surprising. They occupy neighbouring cortex, and functional and structural distinctions between the TPJ and pSTS are not always clear in the literature (e.g. Mars et al., 2011). In terms of overall univariate response magnitude, the two regions do respond quite differently to our shape stimuli, with the pSTS responding robustly, while the TPJ shows a relatively weak response. Indeed, much greater univariate responses in the pSTS than the TPJ were also observed in a recent study by Isik et al. (2017). Despite this univariate difference, we cannot make strong claims for functional separation of these regions based on the present data. However, the relatively stronger classification of interaction vs. non-interaction contrast in the pSTS, along with substantially greater overall univariate response to all the stimuli in the pSTS, might indicate that the two play different roles. If this is true, we speculate that the pSTS may be driven more by visual interaction cues, and the TPJ might play a relatively stronger role in processing explicit inferential information. This interpretation aligns with a trend found in a previous meta-analysis (Schurz et al., 2014): Explicit ToM inferences (e.g. false-belief inferences) that do not require visual action observation tend to activate more posterior regions of the temporo-parietal cortex, whereas tasks that involve extracting intentions from visual actions (e.g. moving shapes) evoke more anterior activation of this area (i.e. pSTS and posterior middle temporal gyrus). Similarly, prior evidence demonstrates that regions of the pSTS respond strongly to a variety of dynamic social visual information (Deen et al., 2015, Lahnakoski et al., 2012), and specifically to visual cues that underlie dynamic interactions, such as correlated motion between moving shapes (Schultz et al., 2005) and action contingencies (Georgescu et al., 2014).

However, a purely visual account the pSTS seems unlikely given top-down modulation of the pSTS when viewing moving shapes (Lee et al., 2012) and apparent sensitivity to the intentional content of visually observed actions (Pelphrey et al., 2004, Saxe et al., 2004). Instead the pSTS may play an intermediate or integrative role between perception and higher-level cognition. Similarly, regions of the STS are demonstrated to integrate multimodal perceptual information (e.g. Beauchamp et al., 2004), whilst one recent study showed a fundamentally integrative neural response to holistic human-object interactions (relative to averaged responses of the constituent parts of these interactions; Baldassano et al., 2017). Understanding interactions undoubtedly requires rapid online integration of multiple dynamic actions between agents, and so the pSTS may play a direct role in the translation of dynamic multi-agent social actions into higher-level social cognition, such as understanding the immediate intentions of the interactors, and the purpose of a social encounter.

It is also important to note that the present research focuses on observed *third-person* interactions, yet these findings may inform other lines of interaction research: For example, Schilbach et al. (2013) have emphasized the importance of extending insights from research focused on third-person social scenes research to second-person interactions (i.e. measuring neural responses of an individual engaged in a real-time interaction with another individual). The extent to which the pSTS is modulated during second-person interactions remains to be fully determined, although pSTS and TPJ modulation has been observed in second-person joint attention tasks (Redcay et al., 2010; i.e. locating a visual target by following the eye-gaze of an individual presented on a screen - ‘joint attention’ > locating the target without the others’ eye gaze - ‘solo attention’). By contrast, second-person interactions that emphasize *social cognition* and do not require *person-perception* (e.g. playing a computer game against an unseen opponent) typically recruit fronto-parietal regions (e.g. superior frontal gyrus and superior parietal gyrus; Decety et al., 2004) that may include mentalizing network regions such as TPJ and precuneus (Tsoi et al., 2016) but no pSTS responses are noted. Further research should aim to clarify the extent to which visual interactive cues are modulated by viewer perspective, along with the contribution of social cognition to responses in these areas.

The present data suggests that the pSTS, and similarly, the TPJ are central to interaction perception and should motivate further research to manipulate specific interaction cues to better characterize responses in these two regions. This is a particularly interesting prospect given a number of recent findings that suggest specialized processing of interactive information; for example, an observed ‘inversion effect’ for interacting dyads, relative to non-interacting dyads (Papeo et al., 2017), perceptual ‘chunking’ of interacting dyads in working memory (Ding et al., 2017), and qualitatively richer neural representations for human-object interactions than for isolated human and object representations averaged together (Baldassano et al., 2017). It remains to be determined what the full set of cues involved in social interaction perception may be, the relative strength of and interactions between these cues, and whether the pSTS is sensitive to such cues in the absence of social information. There is still a great deal of work to be done to build a complete model of the visual perception of interactive behaviour and the brain networks that support such perception, but it is work that we believe will be both fascinating and rewarding.

## Author contributions

K.K and J.W: study design, data-collection, analysis, and writing. P.D: study design, analysis, and manuscript editing.

## Conflicts of interest

None declared.

## Funding

This work has received funding from the European Research Council (ERC) (ERC-2016-StG-716974) under the European Union's Horizon 2020 research and innovation programme (ERC starting grant: Becoming Social).
