# Supplementary material for: Neural responses to visually observed social interactions
Source: Neuropsychologia. 2018 Apr;112:31–9. doi: 10.1016/j.neuropsychologia.2018.02.023 (PMC5899757; doi:10.1016/j.neuropsychologia.2018.02.023)
Supplement: Supplementary file 1 — Supplementary material [file mmc1.docx]

**Supplementary Materials**

**A. Pilot Study**

8 participants were recruited from the Bangor University population (4 females; aged 19 – 29, *M* = 25.2 years, *SD* = 3.55). The stimuli, design, interaction localizer, and fMRI acquisition parameters were almost identical to those used in experiment 2, except that competitive interactions always resulted in failed goal-outcome (e.g. a ball was not successfully pushed into a goal), and cooperative interactions resulted in successful goal outcome (e.g. a ball was successfully pushed into a goal). Participants were scanned whilst watching moving shape videos that depicted either interactive (i.e. competitive or cooperative) or non-interactive scenarios. Percent signal change was extracted from pSTS ROIs for each condition, and entered into t-tests. Significant differences were found in both the interaction > non-interaction contrast (*t*(7) = 3.16, *p*=.008), and the competition > cooperation contrast (*t*(7) = -1.94, *p*=.047), providing preliminary evidence for pSTS sensitivity to moving shape interactions. Additionally, univariate whole-brain analysis revealed strong activation in the right LOTC for the interaction > non-interaction contrast. As this strong peak differed from the experiment 1 results, we included a 6mm sphere centered on the peak of this response as a region of interest in experiment 2.

**B. ROI Lateralization**

We observed some variability in whole-brain peak activation for the interaction localizer task: 10 participants demonstrated peak global activation peak in the right pSTS, whilst the left pSTS contained peak clusters in 6 participants, and non-pSTS peaks were observed in 5 participants. The decision to restrict pSTS ROI analyses to the right hemisphere was motivated by several factors: firstly, that right pSTS activity was observed across all but 2 participants – those with left sided peaks tended to show right activity too; secondly, comparison of classification performance in left lateralized participants – for both left and right pSTS ROIs – demonstrated equivalent classification performance in both ROIs; and thirdly, previous interaction perception studies tend to show right lateralized pSTS activation maxima at group-level. For the TPJ localizer task, most participants showed right-lateralized peak activation with the right TPJ area, and so right TPJ ROIs were selected.

**C. ROI Size and Location**

4 participants had slightly overlapping pSTS and TPJ ROIs (i.e. mean overlap = 9.25 voxels), and so overlapping voxels were removed from the final ROIs. To determine if there was reliable spatial separation between pSTS and TPJ ROIs (see supplementary figure 1), we calculated three paired t-tests (two-tailed) based on the central MNI coordinate in each ROI, for each of the x, y, and z dimensions. The two ROIs did not differ in lateral placement (x-dimension: *t*(14) = 1.69, *p*=.113), but pSTS ROIs were significantly more anterior (y-dimension: *t*(14) = 4.10, *p*=.001) and ventral (z-dimension: *t*(14) = -2.35, *p*=.034) than TPJ ROIs. A paired t-test for the number of voxels in pSTS (*M*  = 102.53, *SD* = 15.16) and TPJ ROIs (*M* = 108.60, *SD* = 14.96), revealed no difference (*t*(14) = -1.08, *p*=.298). In comparison, the LOTC ROI contained 123 voxels.

**D. Motion Energy Analysis**

We conducted a 2 x 2 factorial ANOVA (i.e. interaction and non-interaction as levels of the first factor, with competition and cooperation as levels of the second factor) for stimulus motion energy values for each condition. Neither main effect between interaction and non-interaction (*F*(1,60) = 0.55, p=.462) or main effect of competition and cooperation (*F*(1,60) = 0.03, p=.866), nor interaction term (*F*(1,60) = 0.05, p=.826) was significant, indicating that motion energy did not differ between conditions.

**E. Stimulus Ratings**

An independent group of participants (N=20) likert-rated (i.e. 1 = strongly disagree, 7 = strongly agree) a subset of stimulus videos, and these ratings were entered into three 2x2 ANOVAs. For the question ‘The agents interacted with each other’, a main effect of interaction and non-interaction was observed (*F*(1,19) = 180.96. *p*<.001), but no main effect between competition and cooperation (*F*(1,19) = 0.86. *p*=.366), nor interaction term was significant(*F*(1,17) = 0.00. *p*=.975). For the question ‘The agents were goal-directed’, a main effect of interaction and non-interaction was observed (*F*(1,19) = 115.13. *p*<.001), but no main effect between competition and cooperation (*F*(1,19) = 2.98. *p*=.100), nor interaction term was significant(*F*(1,17) = 2.40. *p*=.138). For the question ‘The agents were alive/animate’, a main effect of interaction and non-interaction was observed (*F*(1,19) = 46.52. *p*<.001), but no main effect between competition and cooperation (*F*(1,19) = 0.94. p=.762), nor interaction term was significant (*F*(1,17) = 0.42. *p*=.524).

**F. Example Moving Shape Stimuli**

Example moving shape stimuli can be viewed via following link:

<https://drive.google.com/open?id=1sjcgU9Wo0XN90MLr_ZrOwmEAdcxNE8PJ>

**Supplementary Figure 1**

*Supplementary figure 1.* Whole-brain activation maps for the interaction localizer task (i.e. point-light human interactions > individual actions). Height threshold = .001(uncorrected), FDR cluster correction (p<.05). A) Localizer data (used to localise pSTS ROIs) in experiment 2 (N=19; peak whole-brain activation is shown at MNI coordinates (x,y,z) 50 -32 -4; B) Localizer data from an independent participant group (not included in either experiment 1 or 2; N=20; peak whole brain activation: 54 -44 16; C) Conjunction analysis between the two datasets in A and B was performed in SPM12 and revealed a single right pSTS cluster (peak MNI coordinates: 52 -44 18; (height threshold = .001(uncorrected), FDR cluster correction (p<.05).


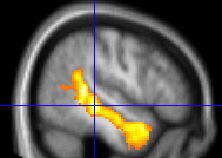

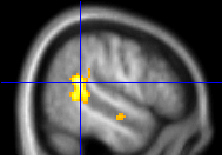

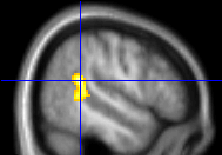


**A**

**B**

**C**

**Supplementary Figure 2**


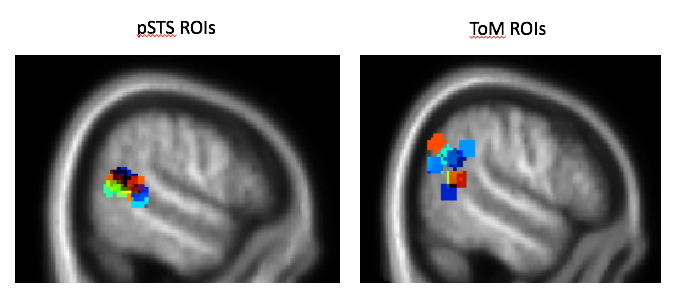


*Supplementary figure 2.* Subject-specific localized ROIs. Left: Right pSTS. Right: Right TPJ.

**Table A**

**Mean centre of ROI coordinates**

| **Dimension** | **pSTS** | **TPJ** |
| --- | --- | --- |
| **X** | 57.23  (5.90) | 53.60  (6.01) |
| **Y** | -46.04  (6.02) | -54.88  (6.55) |
| **Z** | 13.26  (10.39) | 20.51  (8.40) |

*Note.* Values in parentheses are SD.

**Table B**

**Run omissions across participants**

| **Participant** | **Number of Runs Excluded from Analyses** | **Runs** | **Reason for Omission** |
| --- | --- | --- | --- |
| 1 | 1 | 4 | Presentation Script Error |
| 2 | 1 | 4 | Presentation Script Error |
| 3 | 1 | 4 | Presentation Script Error |
| 4 | 1 | 4 | Presentation Script Error |
| 5 | 2 | 4,10 | Presentation Script Error; <75% response accuracy |
| 6 | 1 | 4 | Presentation Script Error |
| 7 | 1 | 5 | >0.5mm Motion Spikes |
| 13 | 3 | 1,2,9 | >0.5mm Motion Spikes (runs 1 and 2);  <75% response accuracy |
| 16 | 1 | 9 | >0.5mm Motion Spikes |
| 17 | 1 | 1 | >0.5mm Motion Spikes |
| 20 | 1 | 8 | >0.5mm Motion Spikes |
| 25 | 1 | 9 | >0.5mm Motion Spikes |

**Table C**

**Participant omissions from Analyses**

| **Participant** | **Analyses Excluded From** | **Reason for Omission** |
| --- | --- | --- |
| 1 | TPJ ROI analyses | Pilot participant – no TPJ localization |
| 2 | TPJ ROI analyses | Pilot participant – no TPJ localization |
| 3 | TPJ ROI analyses | Pilot participant – no TPJ localization |
| 4 | ALL | No button responses |
| 9 | pSTS ROI analyses | No localizable clusters |
| 10 | TPJ ROI analyses | No localizable clusters |
| 11 | ALL | < 50% response accuracy across numerous runs |
| 17 | Supplementary Searchlight Analyses | Frontal lobe signal drop-out due to dental brace |
| 18 | pSTS ROI analyses | No localizable clusters |
